# High Prevalence of Tick-Borne Zoonotic *Rickettsia slovaca* in Ticks from Wild Boars, Northeastern Italy

**DOI:** 10.3390/ani12080967

**Published:** 2022-04-08

**Authors:** Laura Grassi, Maria Luisa Menandro, Rudi Cassini, Alessandra Mondin, Daniela Pasotto, Marika Grillini, Giuseppe Rocca, Michele Drigo

**Affiliations:** 1 Department of Animal Medicine, Production and Health, University of Padova, 35020 Legnaro, Italy; laura.grassi.2@phd.unipd.it (L.G.); rudi.cassini@unipd.it (R.C.); alessandra.mondin@unipd.it (A.M.); daniela.pasotto@unipd.it (D.P.); marika.grillini@phd.unipd.it (M.G.); michele.drigo@unipd.it (M.D.); 2 Euganean Hills Regional Park, 35042 Este, Italy; giuse.rocca@libero.it

**Keywords:** rickettsiosis, *Rickettsia slovaca*, SENLAT, *Dermacentor marginatus*, tick, zoonosis, wild boar, epidemiology

## Abstract

**Simple Summary:**

Tick-borne rickettsioses are emerging diseases that have become widespread in many European countries, particularly in those facing the Mediterranean basin. Although *Rickettsia conorii* was traditionally thought to be the most threatening species, in recent decades, thanks to the improvements in biomolecular tools, other zoonotic species have been identified, such as *Rickettsia slovaca*, the etiological agent of scalp eschar and neck lymphadenopathy after tick bite (SENLAT), as well as other neglected species. These pathogens are present in Italy, but few data are available. This research aimed to improve the epidemiological knowledge of rickettsial infections in tick and wild boar populations in the Euganean Hills Regional Park, an enclosed area in northeastern Italy. Both tick and wild boar blood samples were tested using biomolecular methods to detect and identify *Rickettsia* species. Only ticks tested positive, and *Rickettsia slovaca* was the most frequently detected species, showing a high prevalence, followed by *Rickettsia monacensis* and *Rickettsia helvetica*. These data highlight a non-negligible presence of these pathogens in northern Italy and outline that rickettsial infections deserve further investigation.

**Abstract:**

Tick-borne rickettsiae are emerging pathogens that are becoming widespread in Europe. Rickettsiae are endemic in Italy, but epidemiological data are currently scarce. This study aimed to improve our knowledge about rickettsial infections in tick and wild boar populations. Blood and ticks were collected from 102 wild boars in 2010 and 2018. Ticks were also collected from the vegetation in the area. All of the samples were examined using real-time PCR targeting the *gltA* gene to detect *Rickettsia* DNA. Positivity was confirmed by PCR amplifying the *gltA* and/or *ompB* genes. A total of 254 ticks and 89 blood samples were analyzed. Zoonotic rickettsiae were detected in the ticks but not in the blood samples. *Rickettsia slovaca* (*R. slovaca*) was the most prevalent in ticks and was found in 23.7% of *Dermacentor marginatus* (*D. marginatus*) and in 3.4% of *Ixodes ricinus* (*I. ricinus*). Other zoonotic species were identified, such as *Rickettsia monacensis*, which was detected in 12% of *I. ricinus* ticks, and *Rickettsia helvetica* which was found in 3.4% of questing *I. ricinus* ticks and in 1.1% of *D. marginatus* collected from wild boars. This study highlights a high prevalence of zoonotic rickettsiae, particularly that of *R. slovaca*, in northeastern Italy. As rickettsioses are underreported and underdiagnosed in human medicine, both clinicians and researchers should pay more attention to this topic.

## 1. Introduction

The occurrence of rickettsioses is emerging throughout Europe; it is widespread in many countries, and the human cases are mainly the consequence of tick bites [1,2]. *Rickettsia* spp. are small Gram-negative α-proteobacteria that belong to the Rickettsiaceae family, order Rickettsiales [3]. Tick-borne rickettsiae that cause human diseases in Europe mostly belong to the Spotted Fever Group (SFG). SFG rickettsiae show a distinctive intracellular tropism and mainly infect the peripheral endothelium, and thus clinical manifestations display vascular alterations and lesions, which is the typical symptomatology after infected tick bites [4,5].

Ticks have been described as ancestral hosts for rickettsiae. This ancient co-evolution occurred thanks to a complex interaction between rickettsial bacteria and the immune system of ticks. This allowed tick tissues to become invaded by rickettsiae and ensured the survival of the vector host [6,7]. Indeed, rickettsiae are primarily considered to be symbiotic tick bacteria, and their pathogenic role in mammalian species emerged later.

Thus, the maintenance of this bacterial genus can occur via both transstadial and transovarial routes, and ticks can act both as a reservoir and vector of infection [4,8,9,10]. In addition, ticks may also acquire rickettsiae through sexual transmission or while feeding on a rickettsiemic animal as well as during cofeeding [11].

Moreover, the observed specificity of *Rickettsia* species for a one-tick host genus (or species) is explained by the interference that consists of the inhibition of subsequent rickettsial species from replicating in ticks that are already infected by another *Rickettsia sp.* [12]. Depending on the *Rickettsia* spp. being considered, transmission to vertebrates may be strictly related to one-tick species or to a broader range of competent tick vectors. This can be explained by the high specificity of *Rickettsia* spp. to vector competence together with vertebrate hosts having different susceptibility to infection, meaning that they thus display different epidemiological and clinical features [11]. Indeed, humans who are infected with tick-borne rickettsiae are thought to be accidental hosts, and the onset of disease depends on the pathogenicity of the *Rickettsia* spp. that is involved [13]. In the case of *Rickettsia conorii* (*R. conorii*), the most frequent agent of human rickettsiosis in Europe, transmission is strictly related to the *Rhipicephalus sanguineus* (*R. sanguineus*) tick [1]. The consequent human disease, Mediterranean spotted fever, is reported in many different countries facing the Mediterranean basin, and it has been known for decades [4]. Regarding *Rickettsia slovaca* (*R. slovaca*), an emerging zoonotic species, the main vectorial role is played by the *Dermacentor* tick genus, particularly *Dermacentor marginatus* (*D. marginatus*) and *Dermacentor reticulatus* (*D. reticulatus*) and, *D. marginatus* is the most frequent vector for the human transmission of this pathogen [2,9]. The most frequent clinical manifestation is scalp eschar and neck lymphadenopathy after tick bite (SENLAT) [14]. Human case reports have been described throughout Europe, and especially in Spain, France, Hungary and Portugal [1]. A broad range of *Rickettsia* spp. has been detected in *Ixodes ricinus* (*I. ricinus*), the most widespread hard tick species in European countries. However, this generalist tick mainly appears to be a competent vector for *Rickettsia helvetica* (*R. helvetica*) and *Rickettsia monacensis* (*R. monacensis*) [1]. Although human rickettsioses caused by these two *Rickettsia* spp. are rarely diagnosed, probably due to mild or even absent symptoms, they have been reported in different European countries, including in Italy [15,16].

Currently, tick-borne SFG rickettsioses are considered to be endemic in Italy. Traditionally, most human reports are believed to be caused by *R. conorii*, and cases are mainly reported in Sicily, Sardinia, Calabria and Lazio, where there is an occurrence of about 400 cases/year [17,18,19]. Recently, thanks to advances in biomolecular diagnosis and the increasing amount of attention being paid to the neglected *Rickettsia* spp., other zoonotic rickettsiae have been related to human diseases [19]. For instance, *R. monacensis*, *Rickettsia massiliae*, *Rickettsia aeschlimannii* and *R. slovaca* have been diagnosed in humans from both northern and southern regions in Italy, highlighting the emergence of tick-borne rickettsiosis other than *R. conorii* [14,20,21,22,23]. Among these newly identified species, *R. slovaca* has gained increasing importance, being found with a remarkable prevalence in host-seeking *D. marginatus* in the central regions of the country, along the Tyrrhenian coastline, and up to the Western Alps [17,24]. To date, the central regions of the country are the areas where most of the SENLAT cases have been reported [14,25]. In contrast, northeastern Italy has been poorly investigated not only for *R. slovaca*, but for the occurrence of rickettsiosis in general, although serological studies underline potential undetected circulation [26]. Moreover, non-typhus rickettsiosis is a mandatory notifiable disease in Italy, but it is known to be under-notified and, at the same time, its clinical manifestation is under-ascertained [19].

On these premises, to evaluate *Rickettsia* spp. occurrence and to further improve our knowledge on the spread of these tick-borne pathogens, ticks and wild boar blood samples were collected in 2018 using animals culled within the framework of a depopulation activity, in the Euganean Hills Regional Park, in northeastern Italy. Due to the high prevalence of *R. slovaca* detected in *D. marginatus*, we additionally investigated archived tick and blood samples collected in previous research activities from 2010.

## 2. Materials and Methods

### 2.1. Sampling Site and Specimen Collection

**Figure 1:**
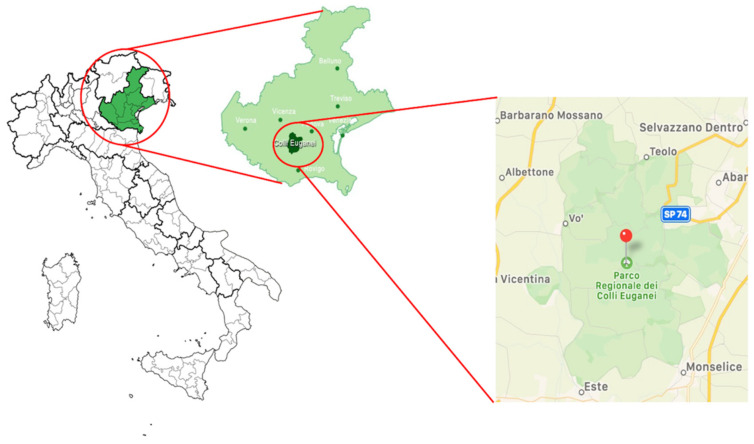
Euganean Hills Regional Park, northeastern Italy.

The investigated area was the Euganean Hills Regional Park, which is located in northeastern Italy and includes 15 municipalities and is characterized by a fragmented environment with woodlands, cultivated fields and peri-urban areas that are strictly interconnected. The Euganean Hills have a maximum altitude of 600 m above sea level. (Figure 1).

Sampling procedures were planned with local practitioners and the park’s rangers. During wild boar culling procedures in 2018 and 2010, which were aimed at controlling their demographic growth, both blood and ticks were collected from this species. Additionally, in 2010, questing ticks were also collected using the dragging method. Blood samples were drawn by cutting the jugular vein, and blood was collected in 9 mL Vacumed^®^ with K3EDTA tubes (FL Medical srl, Torreglia, Padova, Italy), and the samples were refrigerated as soon as possible and brought to the veterinary infectious disease laboratory of the Department of Animal Medicine, Production and Health (University of Padova). Blood samples were divided into 200 µL aliquots and stored at −80 °C until analysis. Ticks were collected in sterile plastic tubes, brought to the laboratory and morphologically identified by means of a stereomicroscope and microscope using identification keys [27,28]. The ticks were also stored at −80 °C until processing.

### 2.2. Biomolecular Analysis

DNA was extracted using the DNeasy blood and tissue kit (QIAGEN GmbH, Hilden, Germany). Immature stages belonging to the same tick species were extracted in pools (up to a maximum of 3 specimens/pool), while adult ticks and blood samples were extracted individually. Internal control and the presence of rickettsial DNA were checked on all of the samples using the Internal Control assay (“Quantinova Pathogen + IC kit”, QIAGEN GmbH, Germany) and real-time PCR targeting a portion of the rickettsial *gltA* gene-based RKND03 system [29], respectively. All of the samples yielding a positive signal during both the Internal Control assay and the rickettsial DNA screening were subsequently confirmed with conventional nested-PCR amplifying a portion of the citrate synthase *gltA* gene (the expected lengths of the primary and nested reaction products were 381 bp and 338 bp, respectively) [30] and/or the *ompB* gene (the expected lengths of the primary and nested reaction products were 381 bp and 338 bp, respectively) [31]. A positive control (DNA extracted from *Rickettsia rickettsii* IFA substrate slides, Fuller Laboratories, California) and a negative control (water instead of DNA) were included in each run. Both strands of the PCR products were sequenced by the Sanger method, and consensus sequences were analyzed using the Basic Local Alignment Search Tool (BLAST) [32] to determine the closest similarity to other *Rickettsia* spp.

### 2.3. Stastical Analysis

Frequency data are reported as counts and are summarized in a table and are categorized based on the year of sampling, the type of sampling, the tick species and stage, tick positivity for rickettsial screening and identified rickettsial species. The frequency of *R. slovaca* positivity among the samples taken between 2010 and 2018 was analyzed using the Chi-square test with the Yates’ correction.

## 3. Results

### 3.1. Sampled Ticks and Wild Boars

Overall, 102 wild boars were included in the study, 88 of which were sampled in 2010 and 14 of which were sampled in 2018. A total of 254 tick samples were obtained from animals and the environment. Most of them were in adult stages, but some immature nymphs (14/254) and larvae (19/254) were also collected from the vegetation. Four different tick species were morphologically identified, namely *D. marginatus* (n = 190, 74.8%), *I. ricinus* (n = 58, 22.8%), *R. sanguineus* (n = 5, 2%) and *Hyalomma marginatum* (*H. marginatum*) (n = 1, 0.4%). Further details on the different tick species, developmental stages and collection methods are provided in Table 1.

Regarding the blood samples, it was not always possible to obtain an adequate specimen, and a total of 89 samples were analyzed (75 in 2010 and 14 in 2018).

### 3.2. Biomolecular Results

The overall prevalence of *Rickettsia* spp. in ticks was 24.8 ± 5.3% (63/254), while all of the wild boar blood samples tested negative. Almost all of the consensus sequences that were analyzed with the BLAST method showed strong sequence similarity (99.41 to 100%) to a specific *Rickettsia* species. In four samples, in which we obtained good-quality sequences of the *ompB* gene only, a clear identification of the *Rickettsia* species was not possible, and therefore, these samples were classified as *Rickesttsia* spp.

The most commonly detected zoonotic species was *R. slovaca,* which was found in 3.4% (2/58) of *I. ricinus* ticks and in 23.7% (45/190) of *D. marginatus* ticks. All of the ticks that were positive for *R. slovaca* were collected from wild boars, and a marked increase in prevalence was observed in the 2018 sampling compared to in the 2010 one, with the prevalence being 61.9% (13/21) and 18.7% (34/182), respectively (X^2^ with Yates’ correction = 17.42; *p* < 0.001). All of the ticks that were collected from the vegetation (n = 51) tested negative for *R. slovaca*. Another zoonotic species that was detected was *R. monacensis*, which was detected in 12% of *I. ricinus* ticks (7/58) that were mainly collected from dragging (6/7), and one was collected from a wild boar. *R. helvetica* was found in 3.4% (2/58) of the questing *I. ricinus* ticks, and in 1.1% (2/190) of *D. marginatus* ticks from wild boars. All of the tested *R. sanguineus* and *H. marginatum* ticks were negative for *Rickettsia* spp. infection. Further details regarding the screening results and species identification are provided in Table 1.

The sequences that were obtained in the present study have been submitted to GenBank and correspond to the codes from OM752204 to OM752300 (Table S1).

## 4. Discussion

Almost all of the ticks that were collected from wild boars were in the adult stages and were males and females, and most of them were *D. marginatus*, findings that are in agreement with other studies [33,34]. Other authors have reported *R. slovaca* infection in *D. marginatus* from wild boars in other Italian regions, with prevalence ranging between 9% and 47.2% [24,33,34]. This broad range may be due to the different geographical areas being investigated, as climate and habitats differ greatly between regions, leading to different epidemiological patterns. Alternatively, host demography can also influence the infection rate in ticks. In our study, the increment in the wild boar population can partially explain the marked increase in prevalence rate of *D. marginatus* ticks between 2010 and 2018.

Single or several ticks were collected from wild boars. Often, several of the *D. marginatus* ticks positive for *R. slovaca* came from the same animal, but due to the convenience sampling performed by the park’s rangers, further conclusions cannot be drawn regarding the cofeeding and the differences in the tick positivity rates with respect to animal infestation.

Interestingly, two *I. ricinus* ticks collected from wild boars also tested positive for *R. slovaca*. These ticks were collected singularly from wild boars, and therefore, they could have been infected at previous stages of development or may have cofed with positive *D. marginatus* ticks. Indeed, to date, *R. slovaca* transmission to humans has only been related to the *Dermacentor* genus [1]. It is noteworthy that all of the *R. slovaca*-positive ticks were collected from wild boars. This could be related to the difficulty of collecting *Dermacentor* spp. via dragging from vegetation compared to collection from animals. Dragging does not seem to be an effective method for *Dermacentor* spp. collection compared to CO_2_ trapping, and harvesting ticks from animals could be easier and more representative of *R. slovaca* circulation.

However, the remarkable prevalence of *R. slovaca* (23.7%) detected in the present study combined with the reports of human bites caused by *D. marginatus* ticks highlights a concrete risk of SENLAT to occur in the investigated area [24,35].

On the other hand, the ticks collected from the vegetation by means of the dragging method were mostly *I. ricinus* ticks, in which other *Rickettsia* spp. such as *R. helvetica* (2/58) and *R. monacensis* (7/58) were detected. The role of *I. ricinus* as an efficient vector of these two species has been previously stated, especially in the Italian regions of northeastern Italy [36,37]. In agreement with previous studies, we reported the presence of *R. helvetica* and *R. monacensis*, which are *Rickettsia* spp. that are typically found in mountainous and inland areas [37,38]. Although these zoonotic species do not seem to be associated with severe disease, evidence of their presence should be of interest, as they have been linked to human disease cases in Italy, as reported by Madeddu et al. [20].

Nevertheless, the high rate of *R. slovaca* infection of *D. marginatus* collected from animals may seem to be in contrast to the absence of this zoonotic pathogen in the wild boar blood samples in our study. In fact, bacteremia is usually only present at low levels and is transient in vertebrates. Rickettsiae are mainly found in the endothelial cells of peripheral blood vessels and, unlike other Rickettsiales such as *Ehrlichia* spp. and *Anaplasma* spp., which circulate in the bloodstream inside monocytic and granulocytic cells, detecting *Rickettsia* spp. in the blood is more challenging [5,17,39]. According to this, the actual role of wild boars has not yet been clarified. Indeed, in other studies, *R. slovaca* DNA has been found in amounts ranging from 1.1% to 11.3% in wild boar spleen, liver and skin biopsies, further complicating our understanding of the concrete involvement of these animals in the rickettsial cycle [24,33,34]. However, many studies agree on their role as indirect amplifiers of the *R. slovaca* infection. In fact, although their role as reservoir species could seem to not be particularly relevant epidemiologically, their involvement in the occurrence of rickettsiosis is due to the fact that they are frequently infested by *D. marginatus* ticks [6].

Therefore, being the preferential feeding hosts for *Dermacentor* ticks, wild boars increase the presence of these vector species together with their associated tick-borne pathogens [33,39]. Besides this, another leading mechanism involved in rickettsiae amplification is cofeeding transmission. Cofeeding is known to occur when the infection diffuses at the local sites of tick bites in highly infested areas, and this is highly probable in wild boars, a finding that is supported by previous studies reporting *R. slovaca*-positive skin biopsies from wild boars [24]. As a consequence of these latter findings, wild boars cannot be excluded from rickettsial epidemiological cycles. These patterns should be kept in mind, considering the high adaptability of wild boars to living in different habitats, including in peri-urban and urban areas, potentially increasing the risk of spreading ticks near human-inhabited areas [6,24].

Unlike the results of our study, other authors found that the prevalence of *R. slovaca* in *D. marginatus* collected from wild boars was similar to the prevalence detected in host-seeking ticks in the same area [34,40], suggesting that wild boars may not play a direct role as amplifiers of rickettsiae.

The hypothesis that may justify the high prevalence found in this study relies on the presence of a reservoir animal on which the immature stages can fed. However, to date, no competent reservoir species for *R. slovaca* has been found. Some authors have suggested that micromammals such as *Apodemus* spp. and *Myodes* spp. may act as potential reservoirs and amplifiers of *R. slovaca* infection, especially when hosting relevant amounts of ticks, although their role has not been clarified [41].

Despite the above-mentioned studies seeming to be in contrast with the findings of this study to some extent, it is clear that *R. slovaca* and tick-borne rickettsioses in general are spreading in many countries, in different animal species—including humans—and, despite an unclear epidemiology, they are spreading widely throughout Europe. In relation to this, recent research conducted by Gomez-Barroso et al. based on Italian data from 2001–2015 highlights that statutorily notified rickettsiosis cases (5.989) comprise almost half of the hospitalized cases (12.032) reporting “spotted fever” or “tick-borne rickettsiosis” as a discharge diagnosis [19]. These data suggest a remarkable underestimation of rickettsiosis occurrence and that the true incidence in the human population is actually unknown. The data reported herein together with this impressive underestimation of rickettsiosis occurrence indicate that the risk of tick-borne rickettsioses is not negligible and deserves more attention.

## 5. Conclusions

This research shows the circulation of zoonotic rickettsiae and the high prevalence of *R. slovaca* in ticks collected from wild boars and vegetation in the Euganean Hills Regional Park, in northeastern Italy. Furthermore, an increase in prevalence of *R. slovaca* has been reported in the 2018 sampling compared to in the 2010 one. The studied area is characterized by a recent increase in these wild animals that may also reach urban areas over time. The large wild boar population, and the associated *D. marginatus* ticks, together with the remarkable presence of outdoor human activities, including agro-pastoral, hunting and touristic activities, as well as the field surveillance carried out by the park’s rangers, represent non-negligible risk factors for tick bite. Thus, the zoonotic rickettsiae detected in ticks analyzed in this study highlight that these emerging pathogens and diseases require more awareness in both human and veterinary medicine.

## Figures and Tables

**Table 1:** Tick sampling data with respect to year and method of sampling, tick species and stage and the results of the real-time PCR screening and *Rickettsia* species detection.

Year	Tick Sampling	Tick Species	Tick Stage	rt-PCR *gltA* Positive/Total	Sequencing (Positive/Total)
2010 (n * = 233)	Dragging (n = 51)	*Ixodes ricinus (I. ricinus)* (n = 47)	Adult (n = 15)	4/15	*Rickettsia monacensis* (*R. monacensis*) (4/4)
			Nymphs (n = 14) (n = 6 s; n = 8 p)	1 s/14	*R. monacensis* (1/3)
				2 p/14	*Rickettsia helvetica* (*R. helvetica*) (2/3)
			Larvae (n = 18) (n = 5 s; n = 13 p)	2 p/18	*R. monacensis* (2/2)
		*Dermacentor marginatus* (*D. marginatus*) (n = 3)	Adult (n = 3)	0/3	-
		*Rhipicephalus sanguineus* (*R. sanguineus*) (n = 1)	Larvae (n = 1)	0/1	-
	Wild boar (n = 182)	*I. ricinus* (n = 9)	Adult (n = 9)	1/9	*R. monacensis* (1/1)
		*D. marginatus* (n = 169)	Adult (n = 169)	38/169	*Rickettsia slovaca* (*R. slovaca*) (34/38)
					*Rickettsia* spp. (4/38)
		*R. sanguineus* (n = 4)	Adult (n = 4)	0/4	-
2018 (n = 21)	Wild boar (n = 21)	*I. ricinus* (n = 2)	Adult (n = 2)	2/2	*R. slovaca* (2/2)
		*D. marginatus* (n = 18)	Adult (n = 18)	13/18	*R. slovaca* (11/13)
					*R. helvetica* (2/13)
		*Hyalomma marginatum* (n = 1)	Adult (n = 1)	0/1	-

n * = number of ticks; s = single tick; p = pool.

## Data Availability

The data generated or analyzed during this study are included in this published case study.

## Supplementary Materials

The following supporting information can be downloaded at: https://www.mdpi.com/article/10.3390/ani12080967/s1, Table S1: Accession numbers of *Rickettsia* spp. sequences deposited into GeneBank.
